# Soft-surface grasping: radular opening in *Aplysia californica*

**DOI:** 10.1242/jeb.191254

**Published:** 2019-08-19

**Authors:** Catherine E. Kehl, Joey Wu, Sisi Lu, David M. Neustadter, Richard F. Drushel, Rebekah K. Smoldt, Hillel J. Chiel

**Affiliations:** 1 Department of Biology, Case Western Reserve University, Cleveland, OH 44106, USA; 2 Cardiac Success Ltd, Yokneam 20692, Israel; 3 Department of Neurosciences, Case Western Reserve University, Cleveland, OH 44106, USA; 4 Department of Biomedical Engineering, Case Western Reserve University, Cleveland, OH 44106, USA

**Keywords:** Feeding, Molluscan behavior, Soft body biomechanics

## Abstract

Grasping soft, irregular material is challenging both for animals and robots. The feeding systems of many animals have adapted to this challenge. In particular, the feeding system of the marine mollusk *Aplysia californica*, a generalist herbivore, allows it to grasp and ingest seaweeds of varying shape, texture and toughness. On the surface of the grasper of *A. californica* is a structure known as the radula, a thin flexible cartilaginous sheet with fine teeth. Previous *in vitro* studies suggested that intrinsic muscles, I7, are responsible for opening the radula. Lesioning I7 *in vivo* does not prevent animals from grasping and ingesting food. New *in vitro* studies demonstrate that a set of fine muscle fibers on the ventral surface of the radula – the sub-radular fibers (SRFs) – mediate opening movements even if the I7 muscles are absent. Both *in vitro* and *in vivo* lesions demonstrate that removing the SRFs leads to profound deficits in radular opening, and significantly reduces feeding efficiency. A theoretical biomechanical analysis of the actions of the SRFs suggests that they induce the radular surface to open around a central crease in the radular surface and to arch the radular surface, allowing it to softly conform to irregular material. A three-dimensional model of the radular surface, based on *in vivo* observations and magnetic resonance imaging of intact animals, provides support for the biomechanical analysis. These results suggest how a soft grasper can work during feeding, and suggest novel designs for artificial soft graspers.

## Abstract (precis)

**Summary:** Newly described muscle fibers control a soft, flexible surface that the marine slug *Aplysia californica* uses to feed on seaweed, suggesting a novel mechanism for constructing a soft grasping device.

## INTRODUCTION

*Aplysia californica* is a generalist herbivore that feeds on red, green and brown seaweeds (Winkler and Dawson, 1963; Kupfermann and Carew, 1974; Carefoot, 1987), which differ biomechanically. The red seaweeds *Laurencia pacifica* and *Gigartina canaliculata* have fern-like branching blades. The brown seaweed *Macrocystis pyrifera* (giant kelp) has blades that form large, leaf-like structures (laminae) supported by air-filled bladders. The green seaweed *Ulva lactuca* (sea lettuce) forms large sheets. Thus, unlike snails that rasp algae, *A. californica*’s feeding apparatus must grasp and tear off pieces of seaweed whose shape, toughness and texture vary significantly, even within a single seaweed (Howells, 1942; Carefoot, 1987). The blades and the stipe supporting them may differ in their dimensions, calcification and chemical defenses (Hay, 1997), posing ongoing biomechanical challenges.

The biomechanics and neural control of feeding in *Aplysia* have been studied intensively (Chiel, 2007). As animals attempt to grasp food, they protract their tongue-like radula (a flexible cartilaginous soft surface covered with fine teeth), closing the radular surface near the peak of protraction (Kupfermann, 1974). A biting behavior is an attempt to grasp food that does not succeed; a swallowing behavior occurs when an animal does successfully grasp food and draws it into the buccal cavity (Kupfermann, 1974). As mechanical load increases, animals increase the force that they exert on seaweed, attempting to break off ingestible pieces (Hurwitz and Susswein, 1992). If an animal swallows material that it cannot ingest, or if the mechanical load becomes excessive, it repositions, releases or ejects the material (rejection), a behavior important for feeding efficiency (Katzoff et al., 2006).

Key muscles and motor neurons controlling the feeding apparatus of *Aplysia* (the buccal mass) have been previously described. Protraction of the grasper, which consists of the radular surface and the underlying musculature of the odontophore (the I4 and I6 muscles, and the cartilaginous, fluid-filled bolsters; Howells, 1942) is mediated by the thin I2 muscle, which is innervated by motor neurons B31, B32, B61 and B62 (Hurwitz et al., 1996). Grasper retraction is mediated by the jaw (the I1/I3 muscles and the underlying jaw cartilage), the contractions of which are induced by the B6, B9, B10 and B3 motor neurons (Morton and Chiel, 1993; Lu et al., 2015), and by the hinge muscle (the interdigitation of the I2 muscle, the I1/I3 muscle, and the major muscle of the odontophore, I4), the force of which is generated by the B7 motor neuron (Sutton et al., 2004a; Ye et al., 2006). Radular closing is mediated by the horseshoe-shaped I4 muscle, which is innervated by the B8a and B8b motor neurons (Morton and Chiel, 1993). *In vitro* experiments suggest that grasper opening is mediated by the I7 muscles that are primarily innervated by the B48 motor neurons (Evans et al., 1996).

Visualizing movements of the internal structures of the feeding apparatus using magnetic resonance imaging (MRI) in intact animals provided important insights into biting, swallowing and rejection (Neustadter et al., 2002a, 2007; Novakovic et al., 2006). A kinematic model based on the MRI studies quantified feeding movements (Neustadter et al., 2002b, 2007), and kinetic models quantified muscle forces during feeding (Sutton et al., 2004b; Novakovic et al., 2006). These models did not incorporate the internal details of the grasper, i.e. the radular surface and the underlying odontophore. Thus, we sought to understand the *in vivo* opening of the radular surface by developing a novel surgical approach to lesion muscles beneath the radular surface, and to create a more detailed three-dimensional model of the process of opening, based in part on the MRI data, using a program that can represent forces acting on a soft surface.

We discovered that I7 plays a role in radular opening, but previously undescribed muscles, the sub-radular fibers (SRFs), also mediate opening and arching of the radular surface, which is critical for the ability of the surface to softly conform to irregular seaweed shapes. Lesions of I7 or of the SRFs significantly reduce radular opening and arching; SRF lesions significantly reduce swallowing efficiency. A theoretical biomechanical analysis predicts how the SRFs may control the radular surface, and a three-dimensional model supports the predictions of the biomechanical analysis. These results have implications for molluscan herbivory, and suggest novel approaches for creating soft-surface graspers.

## MATERIALS AND METHODS

### Animals

Adult *Aplysia californica* J. G. Cooper 1863 (250–400 g) were obtained from South Coast Bio-Marine, San Pedro, CA, USA, and Marinus Scientific, Long Beach, CA, USA. Animals were maintained in aerated 189 liter aquaria containing artificial seawater (Instant Ocean Spectrum Brands, Blacksburg, VA, USA) at a temperature of 16±1°C. Animals were fed 150 cm^2^ of seaweed [dried nori (Sushi-Nori, Nagai Nori Co., Ltd, Torrance, CA, USA) or dulse (Natural Zing LLC, Mount Airy, MD, USA)] every other day. To ensure that animals used for these experiments were healthy, a small piece of seaweed was presented, and animals that generated normal inter-bite intervals (3–5 s; Kupfermann, 1974) were selected for use.

### *In vitro* preparation

**Fig. 1.**
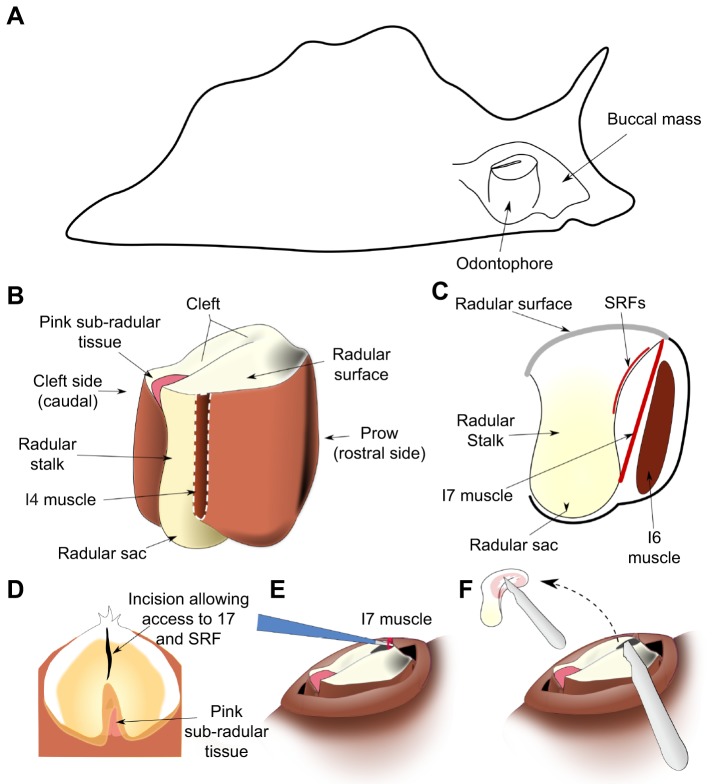
**Anatomy and *in vivo* surgery.** (A) Schematic location of the feeding apparatus (buccal mass) within an *Aplysia californica* body. (B) Schematic external view of radular surface, radular base, radular sac and odontophore muscles. The I4 muscle is removed (white dashed line) to show the radular stalk. (C) Schematic mid-sagittal view, showing locations of muscles I6, I7, and the sub-radular fibers (SRFs) relative to the radular surface. The leaflets of the I5 muscle (not shown in the schematic) insert along the lateral sides of the radular stalk, and do not overlap the SRFs. The SRFs are distinct from the pink sub-radular tissue. (D) Schematic view of the initial surgical incision in the surface of the radula to gain access to the I7 muscle and the SRFs. (E) Schematic view of removal of an I7 muscle. (F) Schematic view of removal of SRFs; the small inset schematically indicates the extent of their removal (approximately two-thirds of the fibers).

The *in vitro* preparation was based on that developed for a prior study of the role of the I7 muscle in opening (Evans et al., 1996; a schematic view of the feeding apparatus within the animal is shown in Fig. 1A, the main muscles of the feeding apparatus are shown in Fig. 1B, and the location of one of the two paired I7 muscles is shown schematically in a lateral cutaway view in Fig. 1C. Animals were anesthetized with an injection of isotonic magnesium chloride equal to their body mass, and the buccal mass was dissected out. An incision through the dorsal midline of the jaws and esophagus exposed the radula and odontophore. The surface of the radula was gently peeled away from the underlying I4/bolster musculature, and the muscles attaching the radular surface to the odontophore were also severed: I6, I7, I8, I9 and I10 (Evans et al., 1996).

The preparation was placed in *Aplysia* saline (460 mmol l^−1^ NaCl, 10 mmol l^−1^ KCl, 22 mmol l^−1^ MgCl_2_, 33 mmol l^−1^ MgSO_4_, 10 mmol l^−1^ CaCl_2_, 10 mmol l^−1^ glucose and 10 mmol l^−1^ MOPS, pH 7.5). For the control group, the non-hydrolyzable cholinergic agent carbachol (Sigma-Aldrich; final concentration 4 mmol l^−1^) was applied to the solution to induce motor movements (Susswein et al., 1996), which were recorded with a video camera. In the experimental preparations, either both I7 muscles, the SRFs, or the I7 muscles and the SRFs were lesioned (see ‘*In vivo* surgery’, below, for further details on lesions of the SRFs). Because the I7 muscle and the SRFs were completely exposed, there was no need to make any incision in the radular surface to access them. Carbachol was added to the bath and the resulting movements were recorded on video.

### Histological analysis of the SRFs

Once the SRFs were identified in the *in vitro* preparations, we examined them histologically (Conn, 1953). All reagents were obtained from Fisher Scientific. Macroscopic observations of the SRFs *in situ* were made by gently brushing them with a dilute solution of Toluidine Blue (0.1% in *Aplysia* saline). To obtain stains of the deeper fibers, the surface was covered in a solution of 0.5% w/v Toluidine Blue in *Aplysia* saline and left to stain for 10 min before washout. Fibers were then photographed using an SMZ-171-TLED Stereo Microscope system with a T2 tablet camera (Motic Optical, North America). To visualize the microscopic structure of the fibers, the buccal mass was dissected out from a 50 g *A. californica* after anesthesia by intra-cavity injection of cold (4°C) 333 mmol l^−1^ aqueous MgCl_2_, then fixed in 10% v/v aqueous phosphate-buffered formalin for 1 week at room temperature (25°C). Excess lip and esophagus tissue were trimmed away, leaving a final specimen approximately 6.6 mm in antero-posterior length. Subsequent processing for histology used standard techniques as described in Humason (1979). Following an overnight rinse in running tap water, the buccal mass was dehydrated in graded ethanols, cleared in xylene, embedded in Paraplast, and serially sectioned perpendicular to the antero-posterior axis at 20 µm. Sections were mounted on Haupt's gelatin-coated slides, dried overnight at 45°C, stained with Mallory's Heidenhain hematoxylin, and coverslipped with Permount. Entire sections were photographed at low power using a Wild M3Z stereo dissection microscope, whereas high-resolution details were photographed using a Zeiss compound microscope.

### *In vivo* surgery

After the animal's mass was measured, animals were anesthetized by injecting an amount of isotonic magnesium chloride (333 mmol l^−1^) equal to approximately one-third of their body mass, and allowed to relax completely. The buccal mass, which can be palpated through the skin when the animal is fully relaxed (Fig. 1A), was gently massaged until it was against the jaws. Gentle pressure was used to push the radular surface and the underlying odontophore through the jaws, taking care to not fully evert the buccal mass, which would cause significant muscle damage, from which the animal would take considerable time to recover (Fig. 1B,C shows the internal anatomy of the muscles surrounding the radular surface). An incision was made in the base of the radula (the side nearest the prow; Fig. 1B) in the mid-sagittal plane (Fig. 1D). Surgical manipulations were applied through this hole in the radular surface. Sham-lesioned animals received this radular incision but no underlying muscles were removed.

To lesion the I7 muscles, the two I7 muscles were identified on either side of the incision, and pulled up through the incision (Fig. 1E). The muscles were carefully disconnected from their upper locations on the underside of the radular surface on either side of the incision, and then pulled upwards so that they could be snipped off at their base near the base of the radular stalk to ensure that they could not re-attach or re-generate during the recovery period. To remove SRFs, a scalpel was inserted through the incision, and the SRFs were carefully removed from the underside of the ventral radular surface around the crease in the radular surface (Fig. 1F; note inset). To avoid excessive damage to the radular surface, only those fibers in the immediate vicinity of the surface incision were removed. Based on post-mortem examination, we estimate that approximately two-thirds of the SRFs were removed. Because the innervation of these muscles has not yet been determined, it was not feasible to denervate them and determine the effects of their passive properties alone.

After surgery, animals were allowed to recover in an isolated aerated small tank suspended within the larger aquarium, and were tested for their ability to feed for up to 1 week after surgery. Animals that did not recover after 1 week were not included in the study.

### Behavioral measurements

Animals were selected for studies based on their motivation to feed. Using the same criterion established by Kupfermann (1974), we selected animals that generated several bites with inter-bite intervals between 3 and 5 s. Bites were obtained after animals had been aroused to feed, and were selected for analysis based on their visibility in the video recordings and the extent to which they were perpendicular to the camera's view.

To the extent possible, blinding was carried out by not informing the investigator who performed the behavioral tests or who analyzed the data which lesion had been done, or whether an animal had only received a sham lesion.

#### Normalized width measurements

**Fig. 2.**
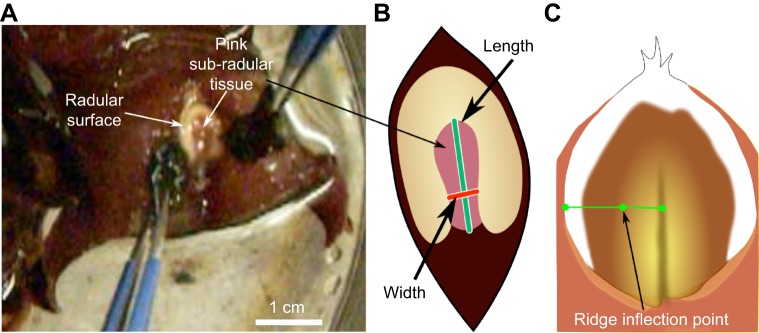
**Measurement of opening width during biting behavior.** (A) Seaweed applied to both sides of the peri-oral zone induced strong, regular bites. (B) Opening width was normalized by the length of the radular cleft. (C) As a proxy for measuring radular surface curvature, the curvature exclusion was determined by measuring the distance from the ridge inflection point to the center of the radula, normalized by the length from edge to center of the radular surface.

Peak opening width was measured in intact animals before surgery and then on the first day animals were able to bite after surgery (up to 1 week post-surgery). Animals were placed in a beaker of artificial seawater and were induced to generate strong bites such that the plane formed by the jaws was parallel to the plane of focus of the video camera by applying small pieces of seaweed to the inner surfaces of the anterior tentacles, near the perioral zone (Fig. 2A). Behavior was recorded on video using a Logitech QuickCam Vision Pro Webcam. Opening width has previously been shown to correlate well with the magnitude of odontophore protraction (Neustadter et al., 2002a). Bites were obtained from each animal prior to and after the lesion (sham lesion, I7 lesion, SRF lesion, or I7 and SRF lesion, referred to as a dual lesion). Measurements of opening were done from the video recording at the peak of bites. The peak of the bite was determined by a frame-by-frame analysis to determine the exact frame at which peak protraction occurred.

To compare animals, measures of opening width were normalized. The width was measured perpendicular to the axis formed by the radular cleft, two-thirds of the distance from the inner surface of the radular base (Fig. 2B). Normalization was performed by taking the ratio of peak opening width to the length (the distance from the inner surface of the radular base to the cleft-side edges of the radular surface; Fig. 2B). The same normalized measure of opening width was used to quantify the openings in the *in vitro* preparation, and in the model.

#### Normalized measurements of radular curvature

During the initial protraction phase of a bite, the radular surface in normal animals shows a clear, gentle curvature, which allows it to conform to the irregular shapes of seaweed; as the radula closes, a sharp ridge forms around the central crease in the radular surface, allowing animals to firmly grasp and pull seaweed inward (see Fig. 2C). A direct measurement of the surface curvature would be extremely difficult, even with two cameras, because the animal has no hard body parts to which cameras could be anchored, and moves considerably during biting behavior. Instead, because the ridge that forms at the peak of protraction during biting completely excludes an area of gentle curvature, and it generates a very characteristic inflection point that can be seen even in single camera views, we chose to measure the percentage of the surface occupied by the ridge as a proxy for the radular surface curvature. The width of the ridge was normalized by the total width of the radula (see Fig. 2C). Animals served as their own controls; four bites were obtained before and after surgery, and the difference in the mean normalized length occupied by the ridge was computed for each animal.

#### Feeding efficiency measurements

To determine the functional effect of lesions on the actual feeding behavior of animals, their ability to swallow seven uniformly sized strips of seaweed (nori, 1×3 cm strips) was measured. As a measure of feeding efficiency, the number of swallows that the animal required to ingest the strip was measured. Animals were used as their own controls, and the difference in the mean number of swallows before and after surgery was computed for each animal, normalized by the mean number of swallows prior to surgery. Although we attempted to quantify feeding efficiency in animals subjected to dual I7 and SRF lesions, their inability to grasp and hold food made this extremely difficult. It should be noted that this indicates that the dual lesions severely reduce feeding efficiency.

### Statistical analyses

For the *in vitro* experiments, in which animals could not serve as their own controls, we used G*power 3.1 (freely available at http://www.gpower.hhu.de; Faul et al., 2009) to determine the size of the groups for ANOVA [power of 0.95, four groups (i.e. sham lesion, I7 lesion, SRF lesion and dual lesion), α value of 0.05, an effect size of 0.1 standard deviations], and found that *N*=6 per group would be sufficient. Bartlett's test showed no significant difference in variance (heteroscedasticity). One-way ANOVA was used to determine overall effects of the lesions, and *post hoc* Tukey tests were performed to determine the significance of the individual lesions.

For the *in vivo* data (change in opening percentage, change in ridge formation, and feeding efficiency), animals served as their own controls. Using G*Power 3.1, we determined that for an α level of 0.05 and an effect size of −0.2±0.1 (determined from pilot studies), we would obtain a power (1−β) of 0.97 for *N*=5, and a power of 0.91 for *N*=4 for a paired *t*-test. To perform ANOVA with the same significance and power levels would have required up to five times as many animals per group according to G*Power 3.1, so we compared magnitudes of effects by computing effect size (Chow et al., 2008): the mean of the differences divided by the standard deviation of the differences.

For the opening percentages and the ridge formation, mean values before and after the lesions (sham lesion, I7 lesion, SRF lesion and dual lesion) were subtracted to obtain a single paired difference in opening percentage for each animal. For the feeding efficiency measures, mean number of swallows (on seven strips of nori) before and after the lesions were subtracted, and divided by the mean number of swallows before the lesion to obtain a single paired normalized feeding efficiency for each animal. For the sham, I7 and SRF lesion groups, we used an *N* of 5. Because of the difficulty of performing combined SRF and I7 lesions, we only used an *N* of 4 for opening measures and an *N* of 3 for ridge formation. Because dual lesion animals fed very poorly, as mentioned above, we did not obtain feeding efficiency measures from them.

Prior to performing a paired *t*-test, we tested for the normality of the differences using the Kolmogorov–Smirnov test, setting the critical value for the test to *P*=0.05. All the data satisfied this test for normality. A one-tailed paired *t*-test was applied with a critical value of 0.05 because we expected that the lesions would decrease the opening percentage. As each animal served as its own control, and the four groups were independent of one another (i.e. sham lesion, I7 lesion, SRF lesion and dual lesion), no corrections were needed for multiple comparisons.

### Model

**Fig. 3.**
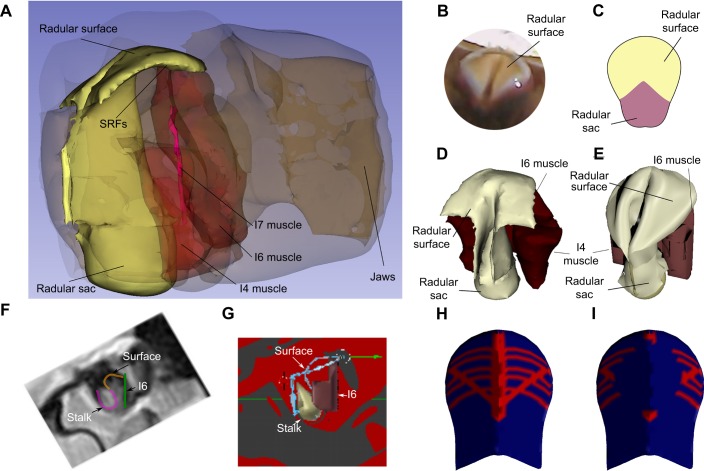
**Soft radular surface model.** (A) Reconstruction of the entire buccal mass based on high-spatial-resolution MRI provided initial resting positions for the muscles beneath the radular surface. (B) A dorsal view of the radular surface. (C) Schematic of the flattened radular surface. (D) Static image of the radular surface and underlying tissues extracted from the high-spatial-resolution MRI model shown in A. (E) Simplification of the structure of the I4/I6 complex (red mesh). (F) Location of structures during biting from a high-temporal-resolution MRI video frame. (G) Radular surface (blue line) is fitted to the model structures (based on MRI, F). (H) Vertex group representing SRFs (see text). (I) Lesion of sub-radular fibers (see Materials and Methods, ‘Model’).

To test biomechanical hypotheses about the functions of the SRFs during opening, and the consequences of lesioning them, a computational model was constructed. First, a detailed, static, three-dimensional model of sub-radular structures thought to contribute to radular movements was made (Fig. 3A; high-spatial-resolution data from Neustadter et al., 2002a; spatial resolution was 0.1×0.1 mm). Anatomical studies allowed determination of the radular shape while intact (Fig. 3B). The initial configuration of the sheet-like radular surface was based on its flattened shape after it was peeled away from the underlying I4 muscles/bolsters (schematically shown in Fig. 3C). The key structures (the I4 muscles and the cartilaginous bolsters within them, and the I6 muscle; see Neustadter et al., 2002b) that contributed to the shape of the surface within the detailed static model are shown in Fig. 3D. These were somewhat simplified for computational efficiency (Fig. 3E; note, for example, the significant simplification of the shape of the I4/I6 complex).

A three-dimensional model that could capture both movements (kinematics) and forces (kinetics) was assembled from these components using the open-source Blender modeling and animation environment (www.blender.org, v.2.77a). The I4 muscles and bolsters and the I6 muscle were represented with a single mesh object (Fig. 3E and G, red; in 3G, the posterior edge of the I4/I6 complex is labeled I6, as that is the location of the I6 muscle). The radular surface (Fig. 3E) and radular sac (Fig. 3D,E) were each represented with separate mesh objects.

When the flattened surface of the radula was attached to the edge of the bolsters, which is anatomically correct (Fig. S1A, leftmost image; Fig. 3G), it was possible to deform the soft surface so that it would accept the radular sac without creating significant collisions between their respective meshes. Insertion of the radular sac mesh is shown in sequence until the surface was complete (rightmost image, Fig. S1E). The final surface configuration (Fig. 3E) was similar to that observed in the animal (Fig. 3B,D).

To ensure the correctness of the kinematics of the underlying structures, which determine the actual deformations of the radular surface during biting, the positions of the radular sac, the margin of the I6 muscle, and the orientation of the I4 muscles and bolsters were determined by carefully analyzing single frames of high-temporal-resolution MRI images (Fig. 3F; mid-sagittal images were acquired every 310 ms at a spatial resolution of 1×1 mm; details of the MRI protocol for obtaining the images are given in Neustadter et al., 2002a, 2007). Once these structures were positioned (Fig. 3G), the edges of the radular surface could be correctly constrained and positioned (Fig. 3G, blue line). Thus, the movements of the underlying structures could now determine the shape of the radular surface. The surface was simulated using a hybrid of the Blender Cloth Simulation Engine and the Bullet Physics Engine (http://bulletphysics.org/wordpress/), so that the shapes assumed by the surface were entirely determined by the movements of the underlying structures and the physical response of the soft surface.

The surface of the radula and the underlying SRFs were represented as a single mesh. All the vertices within the mesh were assigned values indicating the stiffness and ability of edges between them to bend. During the initial assembly phase, the entire mesh was assigned a relatively stiff value to assure that the entire surface would assume an appropriate configuration. After the assembly phase, the mesh as a whole was assigned a relatively low stiffness, so that it could easily bend and fold.

To represent the sub-radular muscle fibers, a group of vertices were defined (see Fig. 3H, indicated in red). Within this vertex group, the values of stiffness could change during the course of a bite. During the time that the SRFs were active, this entire vertex group was assigned high stiffness. During the phase in which the SRFs were passive, these vertices were slightly stiffer than the background value of the rest of the mesh to capture the passive properties of the sub-radular fibers. The timing of activation of the model SRFs was based on the timing of activation determined from EMG measurements of SRFs activity.

To represent the lesion, vertices were removed from the vertex group, corresponding to the removal of the muscle fibers (see Fig. 3I, indicated in red). The timing of activation of the model SRFs was otherwise identical to the control runs of the model.

To test the importance of the timing of the activation of the SRFs, the times that the model SRFs were activated were inverted: during the times that the SRFs would have been active, the values of the vertices were inactive (i.e. only slightly more stiff than the remainder of the mesh), and during the times that they would have been inactive, they were activated (i.e. much stiffer than the rest of the mesh).

A preliminary study using EMG extracellular electrodes in an isolated buccal mass in which I7 and the SRFs were intact indicated that the fibers were activated at times similar to the activation of the I7 muscle (data not shown), so the timing of stiffening corresponded to the timing of activity of the I7 muscle during biting (Evans et al., 1996). Because changing activation of the I7 muscles would alter the movements of the radular stalk, and we have no data on how the radular stalk moves in the absence of the I7 muscle, the model focused solely on how changing the activity of the SRFs altered the opening of the radular surface.

## RESULTS

### *In vitro* experiments on I7 identified a new source of opening movements: SRFs

**Fig. 4.**
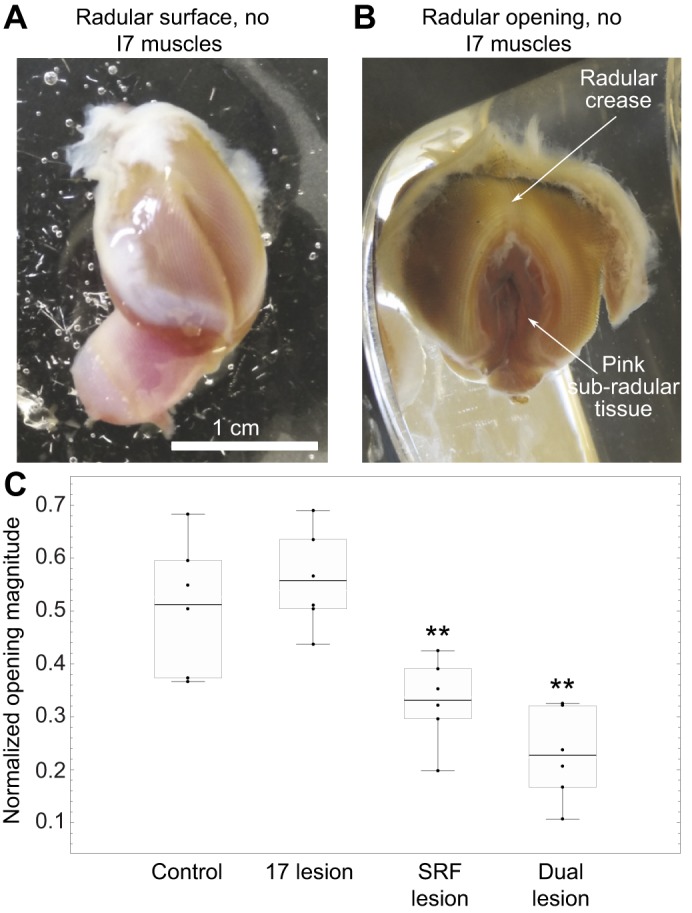
**SRFs contribute to opening *in vitro* in the absence of the I7 muscles.** (A) Reduced preparation for studying opening *in vitro*. The I7 muscles have been removed. (B) Large radular opening induced by carbachol. (C) Comparison of effects of control, I7 lesion, SRF lesion and dual lesions. Overall ANOVA was highly significant (*F*_3,20_=15.07, *P*<0.000023). SRF lesion and dual lesions are significantly different (**P<0.01) when compared with control lesions and I7 lesions; no other differences are significant. Normalized openings were 0.51±0.12, whereas the normalized opening values for the I7 lesioned group were 0.55±0.09 (mean±s.d., *N*=6 preparations for both groups). Normalized opening values for the SRF lesioned group were 0.33±0.08 (*N*=6 preparations; *P*<0.03, Tukey HSD *post hoc* test comparing SRF lesion with control group). The normalized opening values for the dual lesioned group were 0.23±0.09 (*N*=6 preparations; *P*<0.0003, Tukey HSD *post hoc* test comparing dual lesion with control). The middle line in each box represents the mean; top and bottom whiskers correspond to maximum and minimum values; top and bottom of each box correspond to the third and first quartiles, respectively. The original data points are shown as dots. These conventions apply to the box and whisker plots in Fig. 6.

*In vitro* experiments suggested that the I7 muscles were responsible for radular opening (Evans et al., 1996). The reduced preparation in which the I7 muscles were studied consisted of the radular surface dissected free from the underlying musculature (the I4, which contains the bolsters). To understand how the radular surface was controlled by the I7 muscles, we bilaterally lesioned them *in vitro* (see Materials and Methods; Fig. 4A). To our surprise, the *in vitro* preparation continued to show strong openings, both spontaneously and in response to carbachol (Fig. 4B,C; control, I7 lesion; these two groups are not significantly different).

We therefore investigated other muscles that could generate opening, and discovered muscle fibers underlying the surface of the radula, which we termed the SRFs. As these fibers contracted, the radular surface opened. Because these fibers form a thin transparent sheet that might not generate significant force, we tested their role in opening by lesioning them. Lesions of the SRFs led to significant reductions in normalized opening widths *in vitro* in response to carbachol (Fig. 4C, SRF lesion). Lesioning both the SRFs and the I7 muscles led to similar significant deficits in opening (Fig. 4C, dual lesion).

### Anatomy and histology of the SRFs

**Fig. 5.**
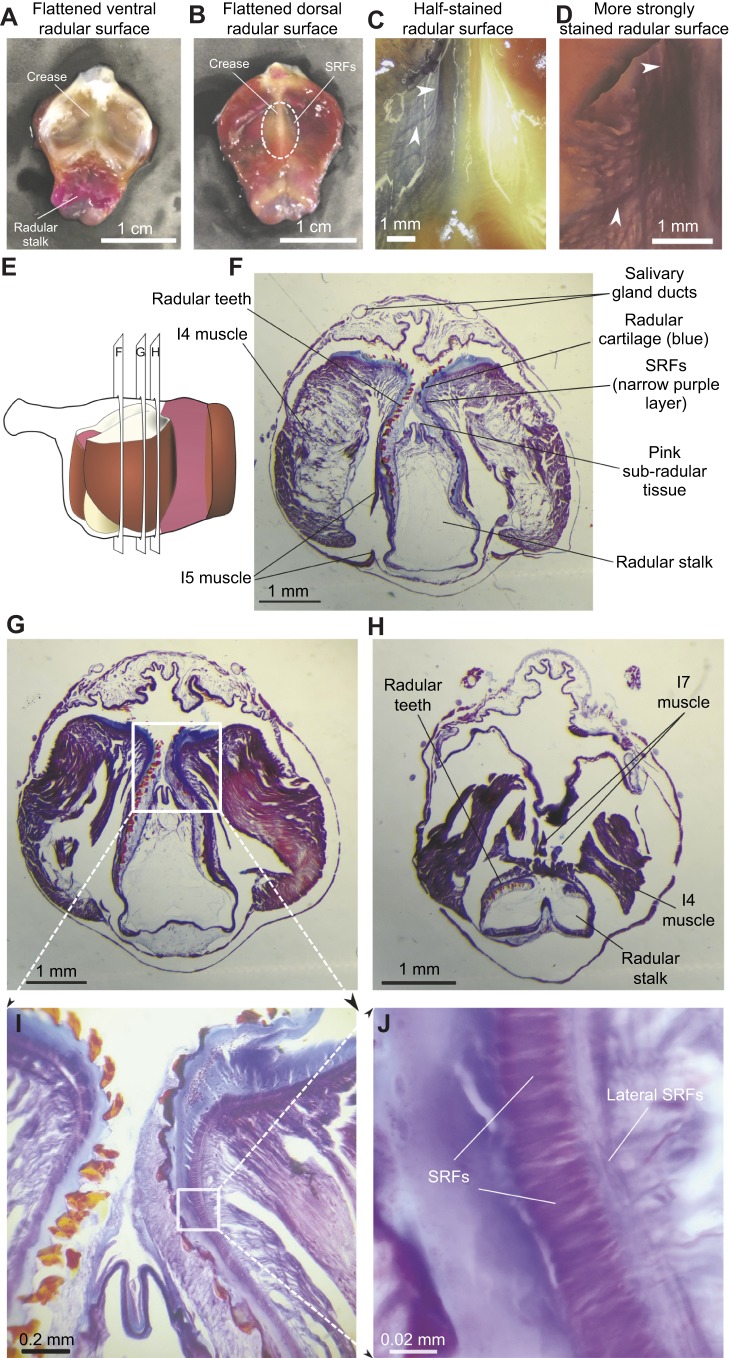
**Anatomy and histology of the SRFs.** (A) Ventral view of flattened radular surface and crease. (B) Dorsal view showing location of SRFs relative to the central crease. (C) Lightly stained ventral surface (see Materials and Methods), showing longitudinal and perpendicular fibers (white arrows). (D) Density of SRFs is clear after heavy staining (see Materials and Methods). (E) Schematic side view of the buccal mass, indicating locations of the sections through the buccal mass shown in F–H. (F) A 20 µm thin section of the buccal mass, showing the very thin layer of SRFs immediately beneath the cartilage of the radula and its teeth. (G) A more anterior section in which the course of the SRFs can be followed more clearly. (H) A still-more anterior section that shows part of the I7 muscles in cross-section. (I) Further magnification of section G (indicated by box), showing the position of the SRFs relative to the cartilage of the radula and its teeth. (J) Magnification of section in I (indicated by box) showing the tight packing of the SRFs, and the course of the perpendicular lateral SRFs.

The SRFs have not been previously described, so we characterized them anatomically and histologically. The reduced preparation could be flattened out (Fig. 5A), and on the ventral side of the radular surface, surrounding a medial crease, fine muscular fibers extended from either side (Fig. 5B). Histological examination (see Materials and Methods) revealed fibers that run longitudinally along the crease (Fig. 5C, light staining; 5D, heavy staining; note white upper arrows), as well as fibers that course both laterally and perpendicular to the crease (Fig. 5C,D; note white lower arrows). Serial sections through the buccal mass (20 µm thickness; Fig. 5E) provided more detailed views. Low-power views of the sections showed a clear region of muscle just beneath the cartilaginous surface of the radula (Fig. 5F,G), and partial sections of the I7 muscles (Fig. 5H). Higher magnifications (Fig. 5I,J) showed a thin layer of tightly packed muscle fibers running longitudinally along the central radular crease, and fibers that also ran perpendicular to the radular crease (Fig. 5J). The muscles showed no striations; rather, they resembled smooth muscle, as observed in many other molluscan species (Hooper and Thuma, 2005; Hooper et al., 2008).

### *In vivo* lesions of the I7 muscles and the SRFs reduce radular opening and arching

**Fig. 6.**
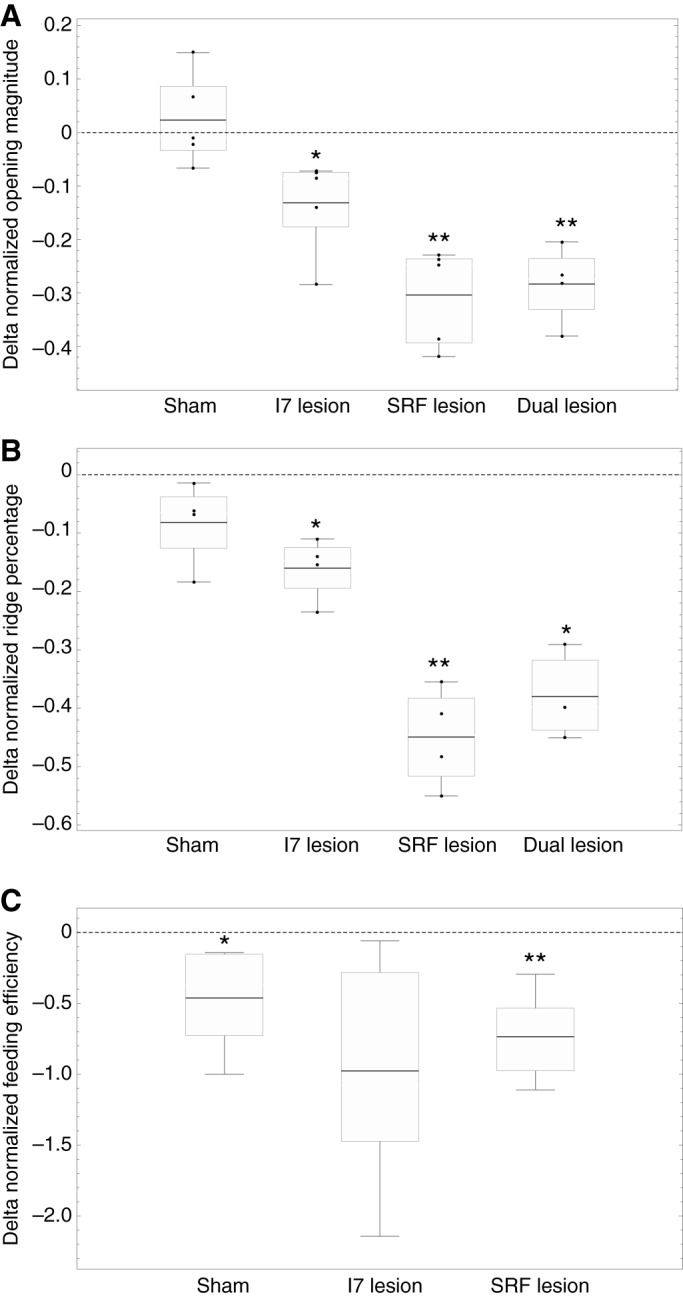
**I7 and SRF lesions reduce radular opening and arching *in vivo*, and SRF lesions reduce feeding efficiency.** (A) Effect of lesions (sham, I7, SRF or dual lesions) on normalized opening magnitude. Data show differences of means for each of five animals before and after lesions (animals served as their own controls; see Materials and Methods). Difference of means pre-sham surgery versus post-sham surgery, 0.024±0.085, mean±s.d., not significant, *N*=5 animals (5 bites from each); effect size=0.28. Pre-I7 lesion versus post-I7 lesion, −0.13±0.09, *P*<0.031, paired *t*-test, *N*=5 animals (5 bites from each); effect size=−1.4. Pre-SRF lesion versus post-SRF lesion, −0.30±0.091, *P*<0.0017, paired *t*-test, *N*=5 animals (5 bites from each); effect size=−3.3. Pre-dual lesions versus post-dual lesions, −0.28±0.073, *P*<0.0044, paired *t*-test, *N*=4 animals (5 bites from each); effect size=−3.8. Note that the effect size of either the SRF or of the dual lesions is larger than the effect size of lesioning I7 alone. (B) Effect of the same lesions on normalized ridge formation, again showing differences of means (*N*=4 animals in sham, I7 and SRF groups, *N*=3 in dual lesion group). Pre-sham surgery versus post-sham surgery, −0.082±0.072, *P*>0.1 (n.s.), *N*=4 animals (4 bites from each); effect size=−1.14. Pre-I7 lesion versus post-I7 lesion, −0.16±0.05, *P*<0.01, paired *t*-test, *N*=4 animals (4 bites from each); effect size=−3.2. SRF lesions alone: −0.45±0.085, *P*<0.0019, paired *t*-test, *N*=4 animals (4 bites from each); effect size=−5.3. Dual lesions: −0.38±0.081, *P*<0.015, paired *t*-test, *N*=3 animals (4 bites from each); effect size=−4.7. Note that the effect size of SRF or dual lesions is larger than the effect size of lesioning I7 alone. (C) Effects of sham, I7 and SRF lesions on feeding efficiency. Differences in mean number of swallows, pre-sham versus post-sham surgery, normalized by pre-sham mean swallows, −0.46±0.36, *P*<0.046, paired *t*-test, *N*=5; effect size=−1.3. Pre-I7 minus post-I7 lesions, −0.98±0.82, *P*=0.056, paired *t*-test, *N*=5; effect size=−1.2. Pre-SRF minus post-SRF lesions, −0.74±0.31, *P*<0.0062, paired *t*-test, *N*=5; effect size=−2.4. Note that the effect size of the SRF lesion is larger than that of either the sham or I7 lesions.

We focused on two behavioral features that are likely to be important to feeding: radular opening and arching. Radular opening was not significantly affected by the sham lesions, but was significantly reduced after bilateral I7 lesions (Fig. 6A, sham, I7 lesion). Opening was significantly reduced by lesions of the SRFs and by lesioning both the SRFs and the I7 muscles (Fig. 6A, SRF lesion, dual lesion). Residual opening is probably due to the remaining SRFs near the base of the radular crease, because only approximately two-thirds of the SRFs were removed (see Materials and Methods). The data support the hypothesis that the SRFs, as well as the I7 muscles, contribute significantly to radular opening.

During early protraction, the radular surface curves gently, allowing it to conform to food. A proxy measure for radular curvature is the formation of the ridge around the crease, which excludes the region of gentle curvature (see Materials and Methods, and below). Sham lesions slightly reduced the area not occupied by the ridge, but these reductions were not significant (Fig. 6B, sham). Bilateral lesions of the I7 muscles significantly reduced the area not occupied by the ridge (Fig. 6B, I7 lesion). Lesions of the SRFs, or of both the I7 muscles and the SRFs, also significantly reduced the area not occupied by the ridge (Fig. 6B, SRF lesion, dual lesion). These data support the hypothesis that the SRFs, along with the I7 muscles, significantly contribute to the arching of the radular surface.

### Feeding efficiency is significantly reduced by SRF lesions

Deficits in grasper opening should reduce the ability to grasp and swallow food so that lesions of the I7 muscles or the SRF could cause swallowing deficits. Animals were fed with seven uniform seaweed strips, and the number of swallows necessary to ingest each strip was measured before and after lesions. Animals were used as their own controls to reduce variability. Animals subjected to both I7 and SRF lesions were difficult to feed; their feeding difficulty clearly indicated qualitatively that the dual lesions were effective in altering feeding. Animals subjected to sham lesions alone showed a significant increase in the number of swallows needed to ingest a strip (Fig. 6C, sham), perhaps because the sham lesion cuts through the region of the SRFs (Fig. 1D). Animals subjected to SRF lesions required significantly larger numbers of swallows to ingest a strip than they did prior to surgery (Fig. 6C, SRF lesion). Although animals receiving I7 muscle lesions showed a trend towards requiring more swallows, the difference was just below the level of significance (Fig. 6C, I7 lesion). Thus, behavioral data support the hypothesis that the SRFs generate opening movements of the radula necessary for normal feeding behavior. The I7 muscles may also contribute to feeding efficiency, but their effects are difficult to separate from the effects of the sham lesions.

### A biomechanical hypothesis for the actions of the SRFs

**Fig. 7.**
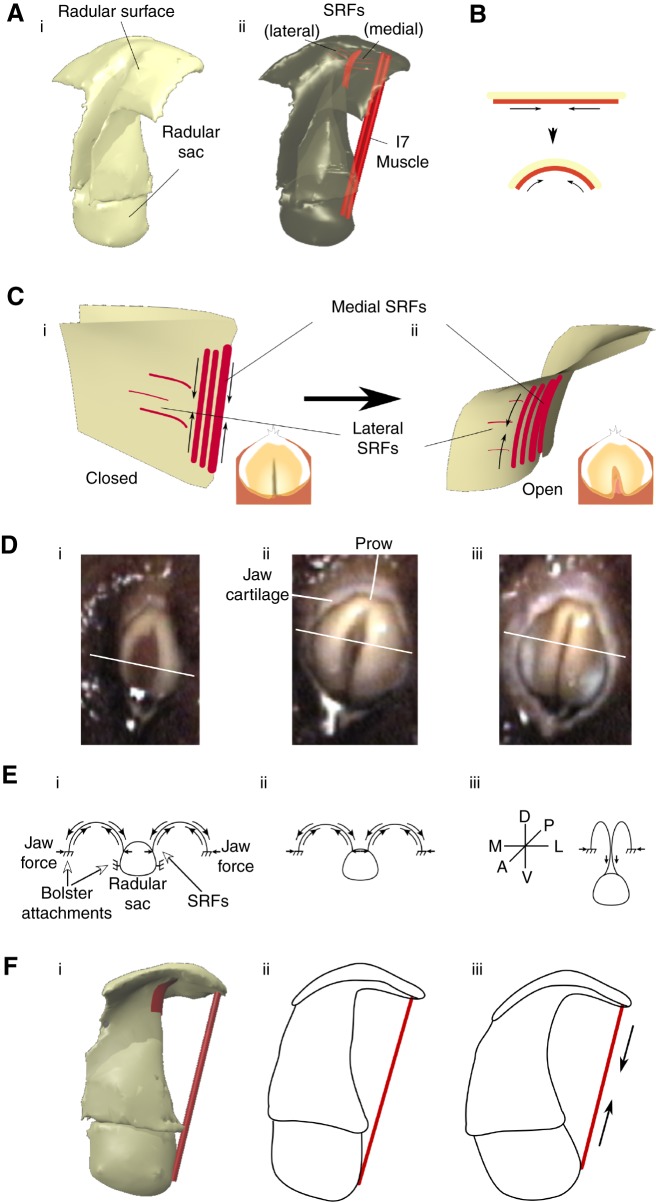
**Biomechanics of the actions of the SRFs and the I7 muscles.** (Ai) Three-dimensional view of radular surface, stalk and sac. (Aii) Location of SRFs and I7 muscles that contribute to opening are highlighted in red. (B) Schematic indicating compressive actions of SRFs that induce outward curvature of radular surface. (C) Stiff crease in radular surface induces SRFs (shown in Ci) to curve radular surface away from crease, causing opening (Cii). Insets show top view of radular surface. (Di) Video frame of early protraction of biting in an intact animal. (Dii) Video frame of peak protraction during the same bite; the opening of the radular halves and their gentle curvature is clearly visible. (Diii) Video frame at the onset of retraction during the same bite. The central stiff ridge is clearly visible. Schematics shown below the video frames (in E) are oriented such that the radular surface is dorsal, the radular sac is ventral, the prow is posterior and the radular cleft is anterior. White lines across the surface of the radula in each video frame (D) indicate the ‘cross-section’ represented by the schematic drawing. Solid arrows represent forces. (Ei) Early protraction. (Eii) Peak protraction. (Eiii) Onset of retraction and ridge formation. See Results, ‘A biomechanical hypothesis for the actions of the SRFs’. (Fi) The location of I7 muscles relative to the radular surface and the radular sac. As the I7 muscles contract (Fii), they pull the radular surface apart and also induce the radular stalk to move towards the radular surface (Fiii).

The surface of the radula is critical during feeding. *Aplysia* grasp seaweed by their foot and anterior tentacles (Kupfermann, 1974), bringing it to the radular surface, which conforms to the irregular shape of seaweed, exerting forces to pull pieces of seaweed inward and break them off. How do the SRFs and the I7 muscles (Fig. 7Ai,ii) contribute to opening the radula and making its surface soft and deformable? A biomechanical analysis suggests testable hypotheses for the muscle functions.

Contraction of the SRFs applies compressive forces to the underside of the radular surface, inducing the upper surface to curve outwards (Fig. 7B). As shown earlier, the radular surface has a central crease that, in the absence of muscular contractions, will tend to induce a fold in the surface (Fig. 5A). Furthermore, the radular surface is quite flexible in response to compressive forces, but very stiff in response to tensile forces, showing almost no elastic expansion when subjected to tensile forces (H.J.C. and C.E.K., unpublished observations). The crease creates an axis of local stiffness within the surface of the radula, so that when the SRFs contract, they cannot deform the tissue along the stiff axis of the crease. Instead, they curve the two surfaces on either side of the crease away from the central crease, inducing opening (Fig. 7Ci,ii). Similarly, the I7 muscles can locally increase the curvature of the radular surface near the prow, applying compressive forces to the underside of the radular surface (Fig. 7Fi–iii), while at the same time moving the radular sac, and thus contribute to the outward curvature of the radular surface, inducing opening around the crease.

The SRFs also contribute to arching radular surface so that it can softly envelop irregularly shaped seaweed. The arching of the yellow radular surface is readily visible from the outside of the animal during a bite (Fig. 7Di,ii). As the animal begins to retract the grasper, the shape of the surface changes, and strongly pinches together to form a ridge (Fig. 7Diii). The ridge makes the radular surface rigid, and this is not due to activation of the I7 muscles, because the I7s are active prior to and after the activation of the closer muscle for the grasper, I4 (see Neustadter et al., 2007, their fig. 14A). Preliminary EMG measurements indicated that the SRFs are active at the same time as the I7 muscles, so they are also not responsible for ridge formation. The fine teeth that line the radular surface face posteriorly, so the ridge helps anchor the teeth within the seaweed, at the same time that the pinched radular surface compresses the grasped seaweed, generating a strong grip that can tear off seaweed pieces.

A biomechanical analysis suggests how the SRFs may generate arching, and how cessation of their activity could lead to the radular ridge. Along its outer edge, the radular surface tightly adheres to the outer edge of the horseshoe-shaped I4 muscle (Fig. 3B); along its inner edge, the radular surface is continuous with the radular stalk (Fig. 3A). To induce opening, both the I6 and the I7 muscles can move the top of the radular surface close to the base of the radular stalk, pulling the radular sac between the halves of the radular surface. The radular surface is then subjected to lateral compressive forces from the surrounding jaw cartilage and medial compressive forces from the radular sac (Fig. 7Ei). When the SRFs are activated, the bottom of the radular surface is subjected to compressive forces, inducing the top of the surface to arch upwards and generate tensile forces that resist the surrounding compressive forces (Fig. 7Eii). To induce closing, the I4 muscle contracts, increasing the distance between the radular surface and the radular sac, which is attached to the base of the I4 muscle through the I8, I9 and I10 muscles (Evans et al., 1996). Initially, this allows the surfaces of the radula to come together medially. As the medial radular surface is pulled ventrally, if the SRFs cease to be active, no forces resist the pinching together of the top of the radular surface, resulting in its compressed, ridge-like appearance (Fig. 7Diii,Eiii). Thus, controlling SRF activation may be important for the control of the soft radular surface.

### Testing SRF function using a three-dimensional model

A model tested the biomechanical role of the SRFs. Using high-spatial-resolution MRI, the internal structures of the grasper were reconstructed. High-temporal-resolution MRI generated movies of the mid-sagittal view of the grasper during swallowing and biting (Neustadter et al., 2002a, 2007), and a three-dimensional kinematic model was previously created to determine the changes in muscle lengths during these movements (Neustadter et al., 2002b, 2007; see Materials and Methods for details of the spatial and temporal resolution of the MRI measurements). We simulated the movements of a soft surface using the open-source Blender animation program, linked to the physics simulator Bullet. The soft surface was anchored to the representations of the I4 muscle, the bolsters and the I6 muscle, and these structures that move the radular surface were induced to follow the same kinematic sequence observed during the MRI movies. Based on microscopic examination of flattened radular surfaces, we set up a coarse mesh to represent the columnar distribution of the teeth on the radular surface. This mesh allowed the surface to fold in ways similar to those observed in the isolated radular surface. The surface was not otherwise controlled by the model.

Elements representing the SRFs beneath the soft radular surface were stiffened in the locations on the radular surface and at the times that the data presented above suggest the fibers should be activated (see Materials and Methods). To capture the effect of lesions of the SRFs, the elements were not stiffened beneath the model radular surface during the movements of the underlying structures.

**Fig. 8.**
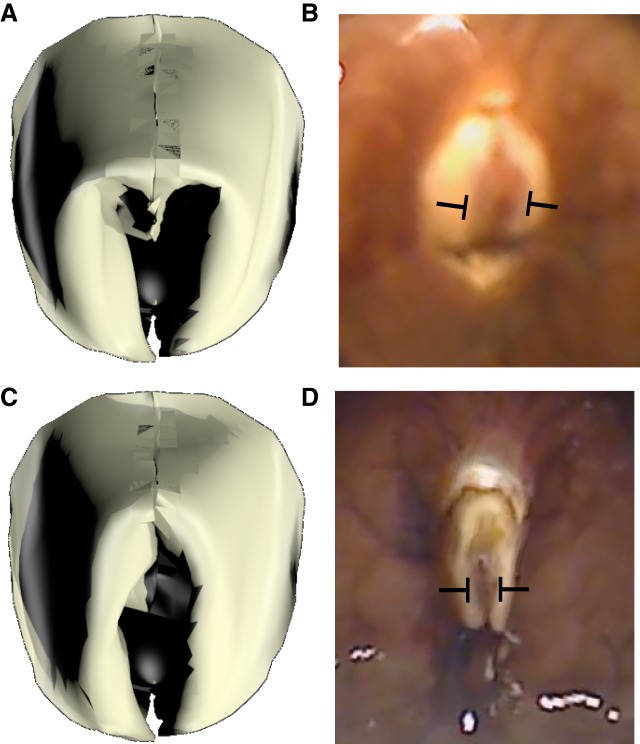
**Model replicates effects of SRF lesion.** (A) Peak protraction, SRFs stiffened; this generates a normal bite. (B) *In vivo* video frame of peak protraction in an unlesioned animal. (C) Peak protraction, SRFs not stiffened; opening is significantly reduced. (D) Video frame of *in vivo* peak bite in the same animal as in B after lesion of SRFs.

When the fibers were stiffened, the surface showed a clear opening and arching of the radular surface (Fig. 8A; compare with the *in vivo* peak protraction, Fig. 8B; also see Movie 1). When they were not stiffened, the surface showed little opening (Fig. 8C; also see Movie 2; compare with the *in vivo* peak protraction of an animal subjected to an SRF lesion, Fig. 8D). We quantified these results by applying the same measurements of normalized opening width to the model, and found that the results were similar to those observed *in vivo* (the difference in the normalized open percentage was −0.46, similar to the maximum effect of SRF lesions, −0.42). Applying the same measurements of change in normalized ridge formation to the model yielded smaller effects than those observed in animals after SRF lesions (a reduction of −0.17; the smallest effect of an SRF lesion was −0.35); this may be due to the inability to fully see the edge of the radular surface in intact animals. Changing the timing of the SRF activation greatly disrupted all aspects of the bite (data not shown; also see Movie 3).

## DISCUSSION

Grasping in *A. californica* requires the radular surface to open and conform to irregular seaweed. Lesions of the I7 muscle lead to deficits in opening and in the ability of the radular surface to arch in intact, behaving animals (Fig. 6A,B), but animals can still bite and swallow. Anatomical and behavioral studies suggest that an additional set of fibers running on the underside of the radular surface on either side of the radular crease, the SRFs (Fig. 5), can induce strong openings *in vitro* in the absence of the I7 muscles (Fig. 4C). Lesions of the SRFs *in vivo* lead to significant deficits in the ability of animals to open and arch the radula (Fig. 6A,B), and reduce feeding efficiency (Fig. 6C). A biomechanical analysis of the SRFs (Fig. 7) suggests that when these fibers shorten along the underside of the radular surface, they induce arching and tension forces in the radular surface that resist compressive forces that would otherwise induce the surface of the radula to pinch together in a hard ridge. A simulation of soft-surface mechanics incorporating the structures that move the radular surface supports the hypotheses that the SRFs control opening and maintain the arching of the radular surface (Fig. 8).

### Relative roles of I7 muscles and the SRFs

What are the relative roles of the I7 muscles and the SRFs in controlling opening of the radula? Lesions of the I7 muscles do induce significant deficits in opening width and arching *in vivo*, whereas lesions of the SRFs cause larger effects on opening and arching (Fig. 6A,B). Lesioning both I7 and the SRFs does not induce an additive deficit in normalized opening width that is significantly larger than the deficit owing to solely lesioning the SRFs (Fig. 6A,B). The observation that lesions of the SRFs also reduce feeding efficiency (Fig. 6C) strongly supports the hypothesis that the SRFs are important for feeding behavior. It is likely that the I7 muscles are important for feeding, although lesions of I7 just failed to reach statistical significance (Fig. 6C). If the I7 muscles play a role in positioning the radular stalk, then lesions of the I7 muscles might contribute to feeding efficiency even if they do not primarily mediate radular opening. Indeed, the I7 muscle is activated during multiple phases of biting and swallowing (Evans et al., 1996 their fig. 4), and thus could affect both opening and closing (see also fig. 14A,B in Neustadter et al., 2007). That I7 could have multiple roles is not surprising. A study of the I5 muscle (also known as the accessory radula closer or ARC muscle) suggested that its effects on the radular surface depended on its mechanical context (Orekhova et al., 2001).

### Radular function and molluscan herbivory

Understanding radular function in *Aplysia* is important for the comparative anatomy of radulae in different molluscan species, and the roles that *Aplysia* play in ocean ecology. Herbivory can significantly affect the distribution of benthic algae and induce a variety of changes in seaweed, including its distribution, chemical defenses and calcification (Duffy and Hay, 1990). An influential study (Steneck and Watling, 1982) examined the relationship between radular morphology and diet, but omitted opisthobranch mollusks because (p. 300) ‘they use their feeding apparatus in a fundamentally different way and thus make comparisons with other herbivorous molluscs difficult’. As described by Howells (1942) and summarized by Carefoot (1987), during feeding, *Aplysia* tear off pieces of seaweed with their radula, and then grind it within their gizzard (Carefoot, 1987). A combined field and experimental study by Pennings (1990) demonstrated that as *Aplysia* grow, their preferred diet can change. Dietary changes may be due to the increased strength of the radula: chopped seaweed (*Codium*) allowed smaller animals to grow faster than the original whole seaweed (Pennings, 1990). A comparative study of several sea hares from the coast of Australia suggested that radular teeth complexity could determine diet: simpler teeth might be associated with a broader diet (e.g. the teeth of *Dolabella auricularis*), whereas more complex teeth might be associated with a more specialized diet (e.g. the teeth of *Stylocheilus striatus*) (Clarke, 2004). A complicating factor, however, is that opisthobranch molluscan morphology may be affected by the diet itself (Trowbridge, 1997). Indeed, a recent study in *Aplysia brasiliana* showed that the molecular biology of related species was nearly identical, but there were significant differences in radular teeth morphology (de Oliveira Saad et al., 2014). Studies of the radula have focused primarily on the teeth covering its surface. To our knowledge, there have been no studies of the dynamic changes of the flexible radular surface during feeding in *A.* *californica*. Understanding the ability of the radular surface to envelop seaweed structures and apply high forces to tear off pieces of seaweed is likely to be important for understanding *Aplysia* herbivory and its implications for the growth and properties of benthic algae.

### Use of small muscles to modulate the functions of large muscles

Small muscles can alter the effects of large muscles in multiple ways: changing the alignment or positioning of large muscles, triggering their actions, steering them or positioning limbs through their passive properties. In the buccal mass, small muscles play a critical role in positioning larger muscles, thus affecting behavior. Relative to the volume of the I4 muscle, the bolsters and the I6 muscle, the other muscles of the grasper are much smaller: the I7 muscles are two thin straps (Fig. 3A); the SRFs are a thin sheet beneath the surface of the radula (Fig. 5); and the I8, I9 and I10 muscles are very small muscles near the base of the radular stalk (Evans et al., 1996). Similarly, the I5 muscle (ARC) is a relatively small muscle that inserts along the radular stalk on one end, and on the outer surface of the I4 muscle at its other end (see fig. 21 of Neustadter et al., 2002b and fig. 2 of Neustadter et al., 2007). Despite their small size, these muscles play critical roles in controlling the radular surface relative to the I4 muscle, the bolsters and the I6 muscle. Similarly, the thin I2 muscle that covers the posterior surface of the buccal mass plays a critical role in protracting the grasper (Hurwitz et al., 1996), which has much larger volume than the I2 muscle. Another example is provided by the very small extrinsic muscles of the buccal mass (E1, E2, E3, E4, E5 and E6) that orient and position the entire buccal mass within the animal's head (Chiel et al., 1986). Despite their small size, lesions of the extrinsic muscles significantly reduce feeding efficiency (Chiel et al., 1986), similar to the effects of lesions of I7 and the SRFs.

Small muscles can affect the actions of much larger muscles through energy release, steering and passive control mechanisms of limb position. In high-speed biological systems, specialized small muscles can rapidly trigger the release of energy stored by much larger, slower muscles (Gronenberg, 1995a,b; Patek et al., 2011; Patek et al., 2006). For example, in the grasshopper, the small flexor tibiae muscle is used as a trigger for releasing energy generated by the much larger extensor tibiae muscle (Heitler, 1974). Similarly, in the alpheid shrimp, the dorsal closer muscle acts as a trigger for their extremely fast claw-closing motion (Kaji et al., 2018). Similar small trigger muscles have also been hypothesized to be critical for the cicada jump (Gorb, 2004), the flea jump (with different small muscle triggers hypothesized in Bennet-Clark and Lucey, 1967 and Rothschild et al., 1972) and the mantis shrimp strike (Burrows, 1969; Patek et al., 2004). Thus, a wide variety of examples have been found in arthropods (reviewed in Gronenberg, 1996 and Patek et al., 2011).

Small muscles can also steer and control much larger muscles. The flight muscles of insects are large, but affect the wings only through indirect linkages; in contrast, small muscles directly insert on skeletal elements of the wing, and thus play critical roles in directing flight (Dickinson and Tu, 1997). Indeed, a recent study has shown that these muscles steadily induce small adjustments throughout flight by their tonic activity, whereas other small muscles can cause large and rapid directional changes through their phasic activity (Lindsay et al., 2017).

Finally, the passive properties of small muscles can affect limb positioning. In the locust, passive forces within the joint can generate flexion, whereas in the stick insect, extension is driven by passive forces, because these muscles are adapted to the strengths of their antagonistic muscles (Ache and Matheson, 2013). Experiments in the locust leg demonstrated that the resting femur–tibia joint position was dependent on the history of prior activation of the large extensor tibiae muscle, again owing to passive forces (Ache and Matheson, 2012). Moreover, passive forces are dominant in small limbs, even in vertebrates such as mice, in which inertial forces are much smaller than passive forces (Hooper et al., 2009).

### Mechanisms by which tongue-like structures acquire food

The arching of the surface by the SRFs during the protraction phase of biting and swallowing increases the likelihood that the radular surface will envelop seaweed, allowing the teeth on the radular surface to penetrate the seaweed as the radula retracts. In turn, the teeth enhance the ability of the radula to pull seaweed into the buccal cavity during the power stroke of swallowing, i.e. retraction (Ye et al., 2006; Lu et al., 2015). Because the teeth face posteriorly (towards the radular cleft), as the radular protracts, the teeth will disengage, allowing the radula to be positioned to grasp the next, more anterior part of the seaweed that the animal is ingesting.

Similar mechanisms in various animals have evolved to allow tongue-like structures to contact food, adhere to it, pull it into the oral cavity, and then release it. The high-viscosity mucus secreted by the chameleon tongue allows the animal to ballistically extend its tongue and rapidly strike moving prey, such as a cricket, and hold onto the prey as it retracts the tongue (Brau et al., 2016), rather than propelling the prey away in response to the impulse force from the tongue. An analysis of the kinematics of frog tongues reveals that mucus and microscopic papillae on the tongue surface act together as a pressure-sensitive adhesive, assuring firm contact without the tongue peeling away from prey until it has been retracted into the oral cavity (Kleinteich and Gorb, 2015). In honeybees, erection of glossal hairs allows the tongue to trap nectar more effectively (Zhu et al., 2016). Cats (Reis et al., 2010) and dogs (Crompton and Musinsky, 2011) lap up fluids using inertial entrainment of the fluid in contact with the dorsal surface of the tongue. Thus, the exact details of the biomechanical solutions vary with the nature of the tongue-like structure, its speed of movement and the properties of the material to be ingested, but each must address the critical problem of adhering to and drawing in food.

### A novel mechanism for soft grasping

Understanding the detailed mechanisms by which soft-bodied animals are able to implement different behaviors has become a greater focus of interest as engineers and roboticists have begun to create artificial soft devices. A recent review focused on robots inspired by the locomotion of worms and caterpillars, as well as by the grasping capabilities of the octopus (Kim et al., 2013). The engineering challenges for control, actuation and fabrication are considerable, but great progress is being made by exploring many materials and control algorithms (Rus and Tolley, 2015; Laschi et al., 2016). An exciting new direction for soft robots and for robotics is the incorporation of biological materials directly into artificial devices – organismal engineering (Webster-Wood et al., 2017).

Creating soft grasping devices has been an area of intense interest for many years, as the rigid graspers characteristic of most robots are unable to handle fragile, soft and crushable materials. Investigators have explored the use of robots with soft fingers (Arimoto et al., 2000; Li and Kao, 2001; Or et al., 2016). A recent study used a topology optimization approach and three-dimensional printing to create a two-‘finger’ soft gripper that could handle a variety of fragile objects of differing sizes (Liu et al., 2018). Taking inspiration from invertebrates, other researchers created a robot that could locomote and grasp underwater, whose body design and function were inspired by the octopus (Cianchetti et al., 2015). A soft grasper was directly inspired by models of the buccal mass of *Aplysia* (Sutton et al., 2004b), and could grasp soft materials and transport them into a lumen using peristalsis (Mangan et al., 2005).

Grasping functions may differ depending on the goals of the organism. Studies of human grasping have distinguished between power grip (between the thumb and all the other fingers) and precision grip (between the index finger and the thumb), whose functions and neural substrates are different (Castiello, 2005). The power grip is used to generate high forces on that object (for example, while climbing a branch) or for wielding a device (such as a hammer) to use it to exert significant forces on other objects. In contrast, the precision grip is used for fine manipulations, or for careful examination of an object. For a feeding system, such as that of *Aplysia*, the animal must also be able to use its grasper for multiple functions: to envelop and attach its radular surface to highly irregular, slippery and mechanically tough foods, and then to exert significant forces to snap off pieces so that the seaweed can be ingested.

### Control of soft surfaces

How can the same soft surface be used for enveloping material, and then for generating the high forces necessary to break off pieces so that it can be swallowed? The biomechanical analysis of the way in which the radular surface can be arched and opened to envelop food, and then closed and retracted to exert strong forces that can break off pieces, is part of a larger area of investigation – controlling the stiffness of soft materials, especially soft surfaces. A recent review focused on activation of soft structures arranged antagonistically, and of actuators whose elastic properties can be changed, to create controllable variable stiffness for robotic devices (Manti et al., 2016).

Other investigators have begun to focus on the control of the stiffness of thin sheets. The surface of the radula is flexible under compression, but does not undergo any significant expansion under tension in any direction (H.J.C. and C.E.K., personal observations). An implication of Gauss's Theorema Egregium (‘remarkable theorem’; Gauss, 1902) is that the intrinsic curvature of the radular surface will not change under deformation, so that if it is bent along one axis (e.g. the crease), the surface perpendicular to this direction will resist bending (become stiff), and this accounts for the ability of the SRFs to induce the sides of the surface on either side of the crease to bend away from the central crease, inducing opening. Similarly, compression of the edges of the radular sheet will induce arching of the surface. These phenomena can be observed in a thin sheet of paper subjected to curvature or to compressive forces along its sides, and analyses of these structures provide new insights into the development of structures with tunable stiffness (Callens and Zadpoor, 2018; Pini et al., 2016). Analysis of the geometric constraints on the responses of thin sheets have now become an important new area of investigation, and may lead to novel ways of deploying materials that are soft and have tunable stiffness (Marder et al., 2007; Li et al., 2012), as well as to the rational design of three-dimensional structures using concepts from origami and kirigami (Callens and Zadpoor, 2018). These concepts may also help clarify other areas of biology, such as the generation of complex morphologies during development depending on whether growth is conformal or generates internal stresses that cause the sheet of cells to assume a different three-dimensional shape (Irvine and Shraiman, 2017). The use of three-dimensional models similar to those developed for the analysis of the SRFs (Fig. 8) may be a useful methodology for investigating these important questions.
